# Atherosclerosis in subjects newly diagnosed with human immunodeficiency virus infection

**DOI:** 10.1042/BSR20180597

**Published:** 2018-07-18

**Authors:** Tatiana V. Kirichenko, Veronika A. Myasoedova, Tatiana E. Shimonova, Alexandra A. Melnichenko, Dmitri Sviridov, Igor A. Sobenin, Alexey I. Mazus, Alexander N. Orekhov, Michael I. Bukrinsky

**Affiliations:** 1 Institute for Atherosclerosis Research, Skolkovo Innovative Center, Moscow, Russia; 2 Unit for Clinical Research in Atherothrombosis, Centro Cardiologico Monzino, Milan, Italy; 3 Moscow City Center for AIDS Prevention and Control, Moscow City Health Department, Moscow, Russia; 4 Southern Federal University, Department of Genetics, Rostov-on-Don, Russia; 5 Institute of General Pathology and Pathophysiology, Russian Academy of Medical Sciences, Moscow, Russia; 6 Baker Heart and Diabetes Institute, Laboratory of Lipoproteins and Atherosclerosis, Melbourne, Australia; 7 Institute of General Pathology and Pathophysiology, Laboratory of Angiopathology, Moscow, Russia; 8 Institute of Experimental Cardiology, National Medical Research Center of Cardiology, Moscow, Russia; 9 The George Washington University School of Medicine and Health Sciences, Department of Microbiology, Immunology and Tropical Medicine, Washington, DC, U.S.A.

**Keywords:** atherosclerosis, anti-retroviral treatment, carotid intima media thickness, cross sectional study, HIV, prospective study

## Abstract

HIV infection is associated with the increased risk of cardiovascular disease (CVD), even in patients successfully treated with the combination antiretroviral therapy (cART). However, the relationship between HIV, cART, and pathogenesis of CVD remains controversial. In the present study, we evaluated the carotid intima–media thickness (CIMT), a surrogate marker of atherosclerosis, in HIV-infected subjects receiving or not receiving cART. One hundred nine newly diagnosed HIV-infected subjects and one hundred nine uninfected age-matched controls (all males) without the history of CVD, hypertension, or diabetes were recruited into the present study. Cross-sectional analysis at baseline (BL) showed significantly increased levels of triglycerides (TG) and decreased levels of high-density lipoprotein (HDL) in HIV-infected subjects, indicating that these risk factors for CVD appeared during the undiagnosed period of HIV infection. Nevertheless, no differences in CIMT were detected between the groups, suggesting that these risk factors were yet to be translated into the clinical disease. The prospective arm of the study, which included 37 HIV-infected and 23 uninfected subjects, showed higher CIMT increase in HIV-infected group than in control group (*P*=0.0063). This difference was significant for both cART-treated (*P*=0.0066) and untreated (*P*=0.0246) subgroups relative to the uninfected subjects, but no difference was found between the HIV-infected subgroups. These results suggest that cART does not reverse the HIV-induced increase of CIMT. The present study demonstrates that the progression of atherosclerosis is accelerated in HIV-infected subjects regardless of treatment.

## Introduction

Introduction of combination antiretroviral treatment (cART) of HIV infection has dramatically reduced mortality and increased the lifespan of HIV-infected subjects. However, co-morbidities that are not directly related to immunodeficiency persist even in the subjects with undetectable viral load in the blood [1]. One such co-morbidity is atherosclerosis and cardiovascular disease (CVD), which has become the major cause of morbidity and mortality of HIV-infected subjects [2]. It is believed that HIV infection and/or its treatment (cART) are associated with the elevated risk of development of atherosclerosis and consequently with increased prevalence of acute and chronic cardiovascular events. However, the contribution of each of these two factors (infection and treatment) to disease pathogenesis remains a debatable issue.

Early generations of antiretroviral regimens, especially those that included protease inhibitors (PI), were associated with dyslipidemia revealed by the high levels of low-density lipoprotein (LDL) and triglycerides (TG) and low high-density lipoprotein (HDL) [3], and may have had other, dyslipidemia-unrelated, pro-atherogenic effects [4,5]. HIV-infected subjects treated with PI-containing cART showed higher prevalence of carotid lesions compared with PI-naive patients or uninfected controls [6]. Current cART regimens, however, are less likely to cause elevation of LDL and TG [7,8], presumably diminishing the pro-atherogenic effects of these treatments. Nevertheless, HIV-associated dyslipidemia is not eliminated in the modern era, as low levels of HDL, a key atheroprotective lipoprotein, persist in both treated and untreated patients [9]. We [10] and others [11] suggested that HIV infection itself, rather than antiretroviral regimens, may be the main contributor to dyslipidemia and elevated risk of atherosclerosis. Indeed, *in vitro* and *in vivo* studies revealed the inhibitory effect of HIV protein Nef on cellular cholesterol transporter ABCA1 and ABCA1-dependent reverse cholesterol transport [12,13], the key step in HDL formation. However, clinical evidence to support the direct role of HIV in pathogenesis of atherosclerosis is inconsistent.

Several studies that demonstrated an increased risk of atherosclerosis in HIV patients [14–17] were cross-sectional or retrospective by design, thus providing only association-based conclusion. A 1-year prospective study reported a faster increase in carotid intima–media thickness (CIMT) in HIV patients on PI-containing regimens compared with HIV-negative individuals, and concluded that HIV infection is an independent risk factor of atherosclerosis [18]. In contrast, our 1-year prospective study did not reveal the CIMT progression in cART-treated or untreated HIV-infected subjects during this short period of time [19]. Another study, a 3-year follow-up of a cohort of HIV-infected subjects (without a control group), demonstrated the progression of CIMT at a higher rate than published values for uninfected subjects, but concluded that ‘traditional cardiovascular risk factors remain the strongest determinants of carotid and coronary atherosclerotic disease progression in HIV-infected patients’ [20]. Other studies demonstrated no impact of HIV infection or non-PI treatment regimens on surrogate markers of atherosclerosis when adjusted for conventional risk factors [21–23], including the two prospective studies [24,25].

In the present study, we assessed atherosclerosis in a cross-sectional study of 109 HIV-infected subjects and 109 uninfected controls, using a surrogate measure of atherosclerosis, CIMT, and followed up some of the participants in these groups over 2 years. We compared groups of HIV-infected patients who remained untreated, those that commenced treatment with cART, and HIV-negative subjects.

## Methods

### Study design and subjects

A 2-year, open-label, case–control, observational study was aimed to provide the prospective data on the progression of atherosclerosis in HIV-infected individuals and the effect of antiretroviral therapy on the dynamics of the CIMT of common carotid arteries as a quantitative surrogate measure of atherosclerosis. The present study was kept in accordance with the Helsinki Declaration of 1975 as revised in 1983. Written informed consent was obtained from each patient included in the study. It was approved by the local ethics committee of the Institute for Atherosclerosis Research, Skolkovo Innovation Center, Moscow, Russia (approval #1-15.10.09). The 109 newly diagnosed HIV-infected male subjects were enrolled at the Moscow City Centre for AIDS Prevention and Control, the Moscow City Health Department. Uninfected male participants (109 subjects) were recruited from the patients attending Moscow municipal outpatient clinic No. 202. All study participants were aged between 22 and 60 years. The exclusion criteria were: history of hypertension, diabetes, CVD, familial hypercholesterolemia, liver dysfunction, body mass index over 27 kg/m^2^. In addition, administration of lipid-lowering medications for 6 months before the enrollment and during the follow-up period was also a criterion for exclusion.

### Measurements

Lipid parameters were measured using the standard laboratory procedures at baseline (BL). Viral load was measured by the quantitative RT-PCR and CD4 T-cell counts – by flow cytometry. Viral load and CD4 T cells were also measured 1 year after the inclusion in the prospective study. The examination of carotid atherosclerosis by IMT was performed using high-resolution B-mode ultrasonography with a linear vascular 7.5 MHz probe on SonoScape S SI-1000 scanner (China). The examination was carried out in a supine position after a 15-min rest and included the scanning of the left and right carotid arteries and the carotid sinus area, keeping a focus on the rear wall of the artery in the three fixed projections – anterolateral, lateral, and posterolateral. Measurements were made at the site of the common carotid artery 10 mm long, opposite to the origin of the carotid sinus. The thickness of the intima–media layer of the posterior wall of the common carotid artery was defined as the distance from the leading edge of the first echogenic zone to the leading edge of the second echogenic zone. The mean of the three measurements (in the anterolateral, lateral, and posterolateral projections) was taken as an integral estimate of CIMT [26]. Scanning was performed by the trained sonographers, unaware of the clinical data. Intima–media thickness measurements were made by the independent certified reader in a blinded manner. IMT measurements were performed by dedicated computer software for IMT analysis, M’Ath (France), which allowed a semiautomatic edge detection of the two echogenic lines defining the boundaries of the intima plus media arterial layers. The use of this program eliminated the errors in measurements that can be made by a technician and provided the necessary quality control. Reproducibility of IMT measurements by this software was assessed according to the protocol of IMPROVE study [27]. The ultrasound examinations of carotid arteries were performed at the BL and at 2 years of follow-up.

### Statistical analysis

Results were expressed as mean CIMT ± S.D. (mm) or as median with interquartile range (IQR). GraphPad Prism 7.0 software was used for statistical analysis. Significance of differences between the groups was evaluated using unpaired Student’s *t* test or Mann–Whitney test, with *P*<0.05 considered significant. Comparison of several groups was done by ANOVA, with Tukey post-hoc analysis.

## Results and discussion

In both HIV-infected and uninfected groups, 38% of the study participants smoked, 37% consumed alcohol regularly, and 48% had family history of CVD. Study participants were characterized by normal values of liver tests (ALT: 40 ± 38 mmol/l and AST: 34 ± 31 mmol/l) and normal blood glucose level. No significant differences between the groups were found at BL in blood pressure, total cholesterol, and LDL cholesterol (LDL-C) (Table 1). However, HIV-infected group had significantly lower levels of HDL cholesterol (HDL-C) than uninfected group (respective mean ± S.D. of 1.12 ± 0.26 compared with 1.37 ± 0.41 mmol/l, *P*=0.001) and higher levels of TG (respective mean ± S.D. of 2.12 ± 1.70 compared with 1.49 ± 0.94 mmol/l, *P*=0.009) (Table 1). High TG and low HDL are the characteristic risk factors of atherosclerosis associated with HIV infection [3]. However, while there was a significant negative correlation between TG and HDL-C levels (Pearson correlation coefficient: −0.320, *P*=0.0008), no correlation was found between CIMT and HDL-C or TG levels, suggesting that these lipids did not significantly contribute to atherosclerosis in this cohort. In fact, CIMT did not significantly correlate with any of the tested parameters. The only borderline correlation (Pearson *r* = 0.126, *P*=0.064) was between CIMT and the number of cigarette packs (20 cigarettes per pack) smoked per week.

**Table 1:** Clinical and laboratory characteristics of study participants

	HIV-infected, *n*=109	Uninfected, *n*=109	*P*-value
Age, years	37.6 (8.5)	36.4 (7.4)	0.24
Systolic BP, mmHg	128 (17)	128 (15)	0.95
Diastolic BP, mmHg	83 (10)	82 (11)	0.39
Smokers, %	51 (5)	35 (8)	0.08
Family history of CVD, %	48 (5)	53 (8)	0.64
ALT, mmol/l	40.3 (37.8)	35.1 (27.0)	0.61
AST, mmol/l	34.3 (30.9)	27.2 (12.2)	0.34
Blood glucose, mmol/l	5.2 (1.00)	5.2 (0.95)	0.81
Total cholesterol, mmol/l	5.2 (1.5)	5.5 (1.0)	0.12
TGs, mmol/l	2.1 (1.7)	1.5 (1.0)	0.007
HDL-C, mmol/l	1.1 (0.3)	1.4 (0.4)	0.001
LDL-C, mmol/l	3.3 (0.9)	3.5 (0.9)	0.27
CIMT, mm	0.645 (0.097)	0.637 (0.099)	0.54
CD4^+^, cells/ml	439 (261)		
Viral load, copies/ml	51; 1602; 26075		

Mean (S.D.) values are shown for all parameters except viral load, for which the 25% percentile, median, and 75% percentile are shown. No significant correlation has been detected between CIMT and any of the measured parameters. BP, blood pressure; ALT, alanine aminotransferase; AST, aspartate aminotransferase.

The subjects recruited for our study were newly diagnosed with HIV infection, but the actual duration of the infection prior to the diagnosis has not been established. Interestingly, no significant difference between the groups was found at BL in CIMT (Table 1), a surrogate marker of subclinical atherosclerosis [17,28]. This result suggests that the duration of undiagnosed infection in the subjects recruited for the present study was insufficient to translate the increased risk factors into a disease, which is consistent with lack of CIMT progression in newly diagnosed HIV-infected subjects (cART-treated or untreated) during a 1-year observation period reported in our previous study [19]. Deterioration of cardiovascular conditions (ultrasonographic data, FMD, inflammation, and cytokine markers) reported previously in treatment-naïve subjects [29] probably occurred after a longer period of uncontrolled infection.

**Figure 1:**
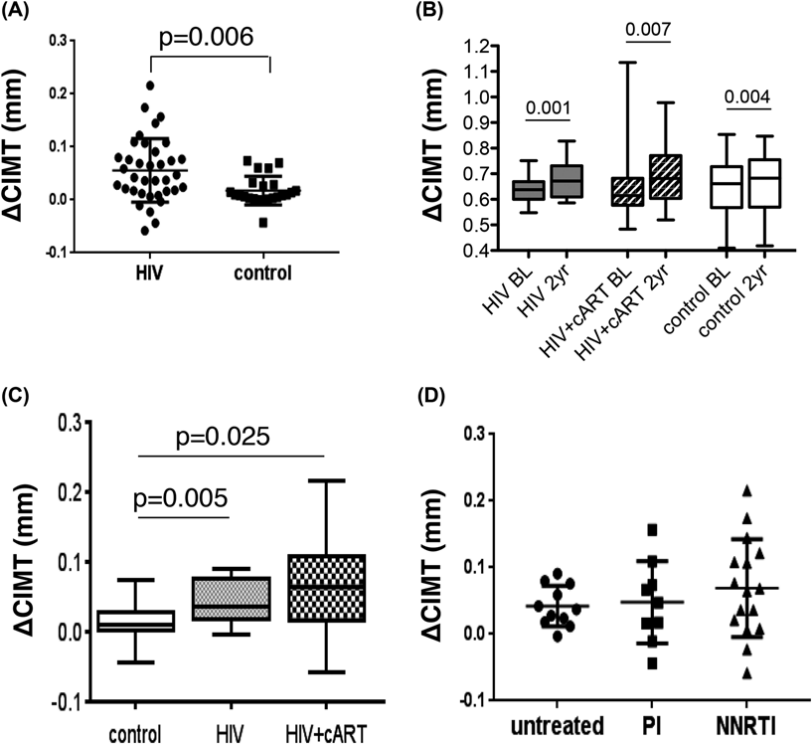
Two-year CIMT increase in HIV-infected and uninfected subjects (**A**) An increase in CIMT during the 2-year follow-up is compared between HIV-infected (both cART-treated and untreated, *n*=36) and uninfected (control, *n*=23) subjects. The plot shows individual values and mean ± S.D. Two-tailed *P*-value was calculated by Student’s *t* test. (**B**) Analysis of CIMT at BL and 2-year time point (2 y) for HIV-infected cART-treated (*n*=25) and untreated (*n*=11) groups, and uninfected (control) group (*n*=23). (**C**) Whisker plot shows median and minimum to maximum range for CIMT change during the 2-year observation period in groups of control (uninfected) subjects (*n*=23), untreated HIV-infected patients (HIV, *n*=11), and cART-treated patients (HIV + cART, *n*=25). Two-tailed *P*-values were calculated by Student’s *t* test. (**D**) CIMT increase is compared between subgroups of PI-treated (*n*=9), NNRTI-treated (*n*=16), and untreated (*n*=11) HIV-infected subjects. Analysis by ANOVA revealed no statistically significant differences between the groups. NNRTI, non-nucleoside reverse transcriptase inhibitor.

Two years after inclusion in the study, the participants underwent ultrasound re-examination of carotid arteries to determine the dynamics of CIMT changes; due to participants’ high dropout rate, only 36 HIV-infected and 23 uninfected subjects completed a 2-year study. Of note, calculation of the sample size for the differences in primary end point, CIMT, to achieve power greater than 0.8 with *P*-value less than 0.05 and expected coefficient of variability of 100%, aiming to detect the minimal physiologically relevant difference in CIMT of 50%, gave a minimal sample size of 22 subjects. The analysis demonstrated a significantly higher CIMT increase in the HIV-infected group (0.056 ± 0.060 mm) relative to the uninfected controls (0.018 ± 0.027 mm) (Figure 1A). To evaluate the effect of cART on CIMT progression, the HIV-infected group was divided in two subgroups; placing subjects who initiated cART (25 subjects) into Group 2, and those who remained cART-naive during the 2-year period – in Group 1 (11 subjects). Of note, according to the Russian National Clinical Recommendations on HIV Diagnostic and Treatment in 2014, when the present study was initiated, the antiretroviral treatment had to be initiated in all the patients with CD4^+^ T cell levels below 350 per μl regardless of HIV load (in 2015, it became 500 cells/μl). For patients with CD4^+^ T-cell levels above those limits, attending physicians decided whether to initiate the treatment. Group 3 (23 subjects) was uninfected controls. Such division compromised the power of analysis, but provided the trends that can be pursued in future studies. BL characteristics for the groups involved in this subanalysis of prospective study are shown in Table 2. Of note, subjects initiating ART tended to have higher viral load at BL than subjects that remained drug-free during the 2-year observation period, justifying the treatment decision. The only statistically significant BL difference between the groups was the level of HDL-C, which was much lower in the group initiating cART (group 2) than in control (uninfected) group 3 (*P*=0.0006). This is consistent with the previously observed effect of HIV infection on HDL-C level and was likely due to a direct influence of HIV on cholesterol efflux and reverse cholesterol transport [9,13]. BL HDL levels in the group that remained cART free were also low, but the difference did not reach the statistical significance (*P*=0.09). This was likely due to the lower viral load in this subgroup of subjects (see Table 2). Again, no differences in CIMT between the groups were found, consistent with the analysis of total recruited population (Table 1). Unfortunately, follow-up measurements of these clinical parameters at the 2-year time point were not available. However, given that no significant correlation was found between CIMT and any of the measured clinical parameters (Table 1), it appeared unlikely that the changes in these parameters could explain differences in CIMT progression (see below).

**Table 2:** BL characteristics of the 2-year prospective study participants

	Group 1, HIV^+^, cART^−^, *n*=11	Group 2, HIV^+^, cART^+^, *n*=25	Group 3, uninfected *n*=23
Age, years	38 (6)	39 (7)	39 (8)
Systolic BP, mmHg	123 (13)	127 (17)	127 (12)
Diastolic BP, mmHg	80 (10)	82 (10)	80 (9)
Total cholesterol, mmol/l	5.1 (1.5)	5.6 (1.9)	5.6 (0.9)
Triglyicerides, mmol/l	2.3 (1.3)	2.3 (1.6)	2.4 (0.6)
HDL-C, mmol/l	1.2 (0.4)	1.1 (0.2)*	1.5 (0.5)
LDL-C, mmol/l	2.8 (0.8)	3.5 (1.1)	3.6 (0.9)
CIMT, mm	0.649 (0.053)	0.646 (0.122)	0.646 (0.112)
CD4, cells/ml	460 (177)	547 (261)	
Viral load, copies/ml	40022 (44361)	226555 (624632)	

Mean (S.D.) values are shown for all parameters. *P=0.0006 vs group 3.

Analysis of CIMT dynamics revealed a significant increase in median CIMT values after 2 years in all the three groups, calculated by paired *t* test (*P*=0.001 for untreated HIV-infected group, *P*=0.007 for treated HIV-infected group, and *P*=0.004 for the control group, Figure 1B). For pairwise comparisons between the groups, we used unpaired *t* test. The CIMT increase in both HIV-infected groups (cART-treated and untreated) were larger than in the control group, and the differences were statistically significant (*P*=0.025 for the untreated HIV-infected group and *P*=0.005 for the treated group, Figure 1C). No statistically significant difference in CIMT increase was observed between untreated and cART-treated HIV-infected groups (*P*=0.677). These results indicate that during the 2 years of observation, atherosclerosis progressed faster in both cART-treated and untreated HIV-infected subjects than in uninfected controls. They also suggest that cART does not slow down the progression of atherosclerosis. The last result is somewhat surprising, but is consistent with previously reported lack of correlation between HIV viral load and atherosclerosis [30,31] and increased risk of atherosclerosis in cART-treated subjects (reviewed in [32]).

Previous studies suggested that the PI-based cART may have pro-atherogenic effect by inducing dyslipidemia [3]. We therefore compared the CIMT increase between the subjects receiving the PI-based cART and those receiving the NNRTI-based treatment (relevant clinical data presented in Table 3). Of note, while in most subjects viral loads were greatly reduced after 2 years of treatment, in most cases to undetectable levels, in two NNRTI-treated subjects (#52 and #60) viral loads were unusually high at a 2-year time point (Table 3). Given that CD4^+^ T-cell counts in these patients were relatively high, it is likely that observed viremia was due to missed medication rather than emergence of drug-resistant virus. We also included a group of untreated HIV-infected subjects in this comparison. Results presented in Figure 1D demonstrate that the CIMT increase was very similar in both cART-treated subgroups, with no statistically significant difference being detected. Moreover, similar to the combined cART-treated group, which did not differ from untreated HIV-infected subgroup (Figure 1C), no significant differences in CIMT increase were detected between untreated (median_ΔCIMT_ = 0.028 mm, IQR: 0.017–0.076) and PI-treated (median_∆CIMT_ = 0.057 mm, IQR −0.004–0.087), or NNRTI-treated (median_ΔCIMT_ = 0.052 mm, IQR: 0.011–0.108) subgroups, although the CIMT increase appeared to be smaller in untreated subjects. This result suggests that PI-based regimen used in the present study does not have any disadvantage in terms of atherosclerosis relative to NNRTI-based regimen. The sample size in this analysis was small (9 PI-treated and 16 NNRTI-treated subjects, Table 3), so these results need further verification.

**Table 3:** cART composition and clinical data for HIV-infected subjects completing the 2-year study

Subject	Drug	cART	CD4^+^ T cells at diagnosis (cells/µl)	HIV VL at diagnosis (copies/ml)	CD4^+^ T cells after 12 months (cells/µl)	HIV VL after 12 months (copies/ml)	ΔCIMT (mm) after 2 years
30	PI	Lamividine/Abacavir, Atazanavir	1298	20	1139	40	−0.044
35	PI	Lamividine, Didanosine, Atazanavir	317	2085	49	513	−0.011
39	PI	Lamividine/Zidovudine, Nelfinavir	34	1818033	244	289	0.157
45	PI	Abacavir, Didanosine, Lopinavir/Ritonavir	250	40	412	20	0.109
53	PI	Lamividine/Zidovudine, Darunavir/Ritonavir	159	20	227	31	0.074
72	PI	Lamividine/Abacavir, Atazanavir	816	20	732	20	0.067
75	PI	Lamividine/Abacavir, Darunavir/Ritonavir	627	68	657	99	0.016
77	PI	Lamividine/Abacavir, Darunavir/Ritonavir	302	20	332	20	0.047
89	PI	Lamividine/Zidovudine, Atazanavir	381	111541	286	40	0.017
3	NNRTI	Lamividine/Abacavir, Evafirenz	432	20	397	20	0.006
9	NNRTI	Lamividine/Abacavir, Evafirenz	837	20	709	20	−0.058
12	NNRTI	Lamividine/Zidovudine, Evafirenz	899	40	685	20	0.069
14	NNRTI	Lamividine/Zidovudine, Evafirenz	952	20	837	20	0.122
15	NNRTI	Lamividine/Abacavir, Evafirenz	729	20	829	20	0.021
16	NNRTI	Lamividine/Abacavir, Evafirenz	454	20	449	20	0.108
20	NNRTI	Lamividine/Zidovudine, Nevirapine	427	20	467	20	0.077
41	NNRTI	Lamividine/Zidovudine, Etravirine	763	20	770	40	0.036
44	NNRTI	Lamividine/Abacavir, Nevirapine	1002	20	952	20	0.107
50	NNRTI	Lamividine/Zidovudine, Evafirenz	32	20	571	40	−0.023
52	NNRTI	Lamividine, Raltegravir, Etravirine	745	20	437	16854	0.216
60	NNRTI	Lamividine/Zidovudine, Etravirine	396	23367	500	14959	0.038
61	NNRTI	Lamividine/Zidovudine, Evafirenz	252	20	252	40	0.065
64	NNRTI	Lamividine/Abacavir, Evafirenz	326	20	491	20	0.145
73	NNRTI	Lamividine/Zidovudine, Nevirapine	252	20	315	20	0.008
78	NNRTI	Lamividine/Abacavir, Evafirenz	811	20	953	20	0.036

Undetectable viral loads were imputed as 20.

No statistically significant correlation between CIMT increase at a 2-year time point and viral load or CD4 T-cell count at BL or at 1-year time point could be found in cART-treated or untreated subjects. Moreover, no significant difference in CIMT change was found between untreated HIV-infected subjects with viral load below the median and those with viral load above the median (not shown). Although, the small number of untreated subjects (*n*=11) precludes reliable conclusion, it appears that viral load does not have a major impact on CIMT increase, consistent with the previous reports [30,31]. In view of previously reported contradictory findings regarding the role of HIV replication in atherosclerosis progression [21–25], it seems reasonable to conclude that the key factor(s) driving progression of atherosclerosis in HIV-infected patients is not virus replication *per se* or immune deficiency. It is more likely that virus-related factors produced during untreated HIV infection, as well as during cART treatment, are the major contributors to atherosclerosis. Such factors include HIV protein Nef, which is released from HIV-infected cells even when virus replication had been suppressed by cART [33], and is known to have pro-atherogenic activity [12,34]. Another such factor is persistent inflammation, which does not subside upon HIV suppression [35] and has a well-established pro-atherogenic effect [36]. These factors have not been analyzed in the present study, so their role remains speculative.

The main limitation of the present study is the small number of subjects completing the 2-year prospective observation period. This was due to high dropout rate, in part owing to the fact that no monetary incentives were offered to the participants. Also, a number of subjects in the untreated group have initiated treatment with cART during the observation period, excluding them from the study. Despite this limitation, analysis performed in the present study supports an important conclusion that atherosclerosis progression is accelerated in HIV-infected subjects, and this acceleration appears to be similar in patients treated and untreated with cART. This outcome apparently contradicts our previously published report [19], which did not find atherosclerosis progression in HIV-infected subjects in a 1-year prospective study. A possible explanation may be that CIMT changes occurring in 1 year were too small to be detected, especially given that subjects in our 1-year study were recruited in Australia and had substantially lower viral load (and probably other virus-related pro-atherogenic factors described above, such as circulating Nef and inflammation) than subjects in the present study (mean BL levels were under 1 × 10^5^ copies/ml for Australian cohort and approximately 3 × 10^5^ for the Russian patients). Faster progression of atherosclerosis detected by CIMT changes in HIV-infected subjects than in uninfected controls is consistent with the results of cross-sectional studies that reported higher CIMT in HIV-infected cohorts [15–17]. Our study did not reveal differences in CIMT progression between cART-treated and untreated groups, similar to another 2-year prospective study that also did not find statistically significant differences between these groups [37]. An interesting observation of our study is the lack of correlation between CIMT progression and viral load or CD4 T-cell counts, even in untreated group, suggesting that other factors, such as Nef and inflammation, may drive atherosclerosis progression in HIV-infected subjects. Overall, the present study supports the notion that HIV infection is an independent risk factor for atherosclerosis.

## Abbreviations

ABCA1: ATP-binding cassette A1
ALT: alanine aminotransferase
AST: aspartate aminotransferase
BL: baseline
cART: combination antiretroviral therapy/treatment
CIMT: carotid intima–media thickness
CVD: cardiovascular disease
FMD: flow-mediated dilation
HDL: high-density lipoprotein
HDL-C: HDL cholesterol
IMT: intima media thickness
IQR: interquartile range
LDL: low-density lipoprotein
LDL-C: LDL cholesterol
NNRTI: non-nucleoside reverse transcriptase inhibitor
NRTI: nucleoside reverse transcriptase inhibitor
PI: protease inhibitor
RT-PCR: reverse transcription-polymerase chase reaction
TG: triglyceride
